# Psychological effects of cognitive behavioral therapy on internet addiction in adolescents: A systematic review protocol

**DOI:** 10.1097/MD.0000000000018456

**Published:** 2020-01-24

**Authors:** Ying-ying Zhang, Jian-ji Chen, Hai Ye, Lupe Volantin

**Affiliations:** a School of Teacher Education, Taizhou University, Linhai, Zhejiang; b College of Music and Dance, Yichun University, Yichun, Jiangxi, China; c School of Social and Community Medicine, University of Bristol, Bristol, UK.

**Keywords:** adolescents, anxiety, cognitive behavioral therapy, depression, internet addiction

## Abstract

**Background:**

In this study, we aim to assess the psychological effects of cognitive behavioral therapy (CBT) on internet addiction (IA) in adolescents.

**Methods:**

This study will search the following databases of Cochrane Library, PUBMED, EMBASE, Scopus, Web of Science, PsycINFO, Chinese Biomedical Literature Database, and China National Knowledge Infrastructure. All these electronic databases will be searched from inception to the September 30, 2019 without any language limitation. Two authors will conduct study selection, data extraction, and study quality assessment, respectively. Any disagreements between 2 authors will be solved by a third author through discussion. Statistical analysis will be performed using RevMan 5.3 software.

**Results:**

This study will investigate the psychological effects of CBT on IA in adolescents by measuring psychopathological symptoms, depression, anxiety, time spent on the internet (hours/day), and health-related quality of life.

**Conclusion:**

This study summarizes current evidence of CBT on IA in adolescents and may provide guidance for both intervention and future researches.

**PROSPERO registration number**: PROSPERO CRD42019153290.

## 1 Introduction

The Internet has provided a new communication medium and has been widely used worldwide.^[1,2]^ However, heavy internet use has been associated with serious side effects and can cause internet addiction (IA).^[3–5]^ Serious problems associated with IA among adolescents consist of refusal to attend school, cognitive problem, physical or psychological disorders, such as anxiety and depression.^[6–13]^ It has been reported that overall prevalence of IA ranges from 2.6% to 10.9% in Western and Northern Europe, and Middle East, respectively.^[14]^ It is about 4.4% of adolescents in 11 European countries.^[15]^ If it has not been treated fairly well, it may cause serve psychological disorders. Thus, it is very important to prevent and treat such condition among adolescents population.

Presently, several interventions have been proposed for the managements of psychological disorder adolescents with IA, such as cognitive behavioral therapy (CBT).^[16–19]^ A variety of studies have reported that CBT can be utilized for the management of adolescents with IA.^[18–23]^ However, their results are still contradictory, and no systematic study has explored to check the psychological effects in adolescents with IA. Therefore, this study will investigate the psychological effects of CBT in adolescents with IA.

## 2 Methods

### 2.1 Study registration

We have registered this study protocol on PROSPERO (CRD42019153290). We will report this study based on the Cochrane Handbook for Systematic Reviews of Interventions and the Preferred Reporting Items for Systematic Reviews and Meta-Analysis Protocol statement guidelines.^[24]^

### 2.2 Criteria for study selection

#### 2.2.1 Study types

We will include randomized controlled trials (RCTs) that measure the psychological effects of CBT on IA in adolescents. Any nonclinical studies, uncontrolled trials, and non-RCTs will be excluded.

#### 2.2.2 Participant types

Any adolescents who are diagnosed as IA will be included in this study, regardless their race and gender.

#### 2.2.3 Intervention types

In the experimental group, any RCTs that utilize CBT as only intervention will be included in this study.

In the control group, participants who received any treatments except CBT will be included.

#### 2.2.4 Outcome types

The primary outcomes include depression (checked by any scales, such as Self-Rating Depression Scale), and anxiety (assessed by Self-Rating Anxiety Scale or any other relevant scales).

The secondary outcomes are psychopathological symptoms (measured by Symptom Check List-90 or other scales), time spent on the internet (hours/day), and health-related quality of life (evaluated by Young Internet Addiction Scale or associated tools).

### 2.3 Search methods

We will search the following electronic databases: Cochrane Library, PUBMED, EMBASE, Scopus, Web of Science, PsycINFO, Chinese Biomedical Literature Database, and China National Knowledge Infrastructure. We will search all these electronic databases from their inception up to the September 30, 2019 without any language limitation. The search strategy sample for Cochrane Library is presented in Table 1. Similar search strategies will be adapted and applied to the other electronic databases.

**Table 1:**
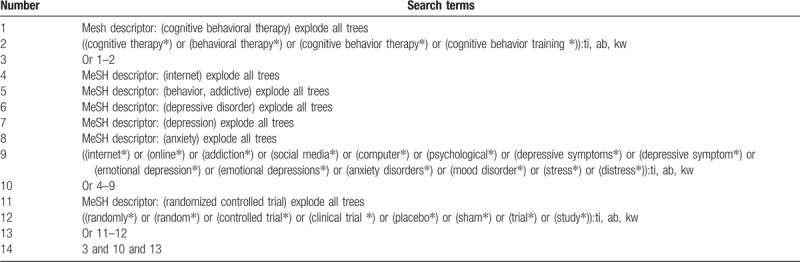
Search strategy utilized for Cochrane Library.

In addition to the electronic databases, we will also search conference abstracts, dissertations, and reference lists of relevant reviews.

### 2.4 Data collection

#### 2.4.1 Study selection

Two authors will independently undertake selection of studies based on the predefined inclusion and exclusion criteria. First, the titles and abstracts of all records will be scanned, and papers that fail to meet eligibility criteria will be excluded. Second, we will obtain full papers of the remaining records, and will evaluate them against inclusion criteria to determine their eligibility for inclusion. We will present the whole process of study selection in the flowchart. Any disagreements between 2 authors will be solved through a consensus-based decision with the help of a third author if necessary.

#### 2.4.2 Data extraction process

Two authors will independently extract the data from each eligible study using a standardized data extraction sheet. Any discrepancies between 2 authors will be resolved by a third author through discussion. We will extract the following data: title, first author, publication time, baseline characteristics, diagnostic criteria, inclusion and exclusion criteria, sample size, study setting, study methods, details of treatment and controls, all outcome measurements, safety, and any other relevant information.

#### 2.4.3 Missing data management

When unclear or insufficient data is identified, we will contact primary authors to require them. If we cannot obtain such data, we will analyze available data and will discuss its possible impacts.

### 2.5 Study quality assessment

Study quality will be evaluated using Cochrane Risk of Bias Tool, which is designed to assess RCTs. It has 7 domains and each one is further assessed as high, unclear or low risk of bias. Two authors will assess each eligible study, respectively. Any discrepancies will be solved by a third author through discussion.

### 2.6 Measurement of treatment effect

Continuous data will be calculated as mean difference or standardized mean difference and 95% confidence intervals. Dichotomous data will be expressed as risk ratio and 95% confidence intervals.

### 2.7 Assessment of heterogeneity

The test of *I*^*2*^ will be used to detect heterogeneity among included studies. An acceptable heterogeneity is defined when *I*^2^ ≤50, while substantial heterogeneity is considered when *I*^2^ > 50%.

### 2.8 Statistical analysis

#### 2.8.1 Data synthesis

RevMan 5.3 software is used for statistical analysis. We will synthesize the data using a fixed-effects model if acceptable heterogeneity identified; and we will perform meta-analysis. We will use a random-effects model if substantial heterogeneity is found. In addition, we will also apply subgroup analysis. If there is still significant heterogeneity after subgroup analysis, we will report narrative summary based on the study characteristics, participant characteristics, such as gender and ethnicity, and findings by intervention types.

#### 2.8.2 Additional analysis

We will perform subgroup analysis to explore reasons for significant heterogeneity according to the different types of study quality, treatments, controls, and outcome measurements.

In addition, we will also conduct sensitivity analysis to check robustness of outcome results by removing low-quality studies.

#### 2.8.3 Reporting bias

If at least 10 eligible RCTs are included, Funnel plot^[25]^ and Egger regression test^[26]^ will be checked to identify if there are any reporting bias.

### 2.9 Ethics and dissemination

This study will not use individual patient data, so it does not need ethical approval. Our results are expected to be submitted for a peer-review publication.

## 3 Discussion

This study will provide an up-to-date assessment of CBT used for the management of IA in adolescents. To our best knowledge, this study will specifically focus solely on investigating the psychological effects of CBT management for adolescents with IA. Although several previous studies have reported to treat IA in adolescents effectively using CBT, there are still inconsistent conclusions regarding this topic. Therefore, this study will systematically investigate the psychological effects of CBT in adolescents with IA. The results of this study will provide recommendation for the treatment of adolescents with IA using CBT.

## Author contributions

**Conceptualization:** Ying-ying Zhang, Jian-ji Chen, Hai Ye.

**Data curation:** Ying-ying Zhang, Jian-ji Chen, Lupe Volantin.

**Formal analysis:** Ying-ying Zhang, Hai Ye.

**Funding acquisition:** Ying-ying Zhang.

**Investigation:** Ying-ying Zhang, Lupe Volantin.

**Methodology:** Jian-ji Chen, Hai Ye.

**Project administration:** Ying-ying Zhang.

**Resources:** Jian-ji Chen, Hai Ye, Lupe Volantin.

**Software:** Jian-ji Chen, Hai Ye, Lupe Volantin.

**Supervision:** Ying-ying Zhang.

**Validation:** Ying-ying Zhang, Hai Ye, Lupe Volantin.

**Visualization:** Ying-ying Zhang, Jian-ji Chen, Lupe Volantin.

**Writing – original draft:** Ying-ying Zhang, Jian-ji Chen, Hai Ye.

**Writing – review & editing:** Ying-ying Zhang, Jian-ji Chen, Lupe Volantin.
